# Demographic determinants of happiness in Andalusia: insights from the 2022 social survey data

**DOI:** 10.3389/fpubh.2024.1338494

**Published:** 2024-05-02

**Authors:** Antonio Matas-Terrón, Carmen Sánchez Barroso, José M. Matas-Terrón

**Affiliations:** ^1^University of Málaga, Málaga, Spain; ^2^Amaveca Health Hospital, Lucena, Spain; ^3^Andalusian Health Service, Málaga, Spain

**Keywords:** happiness, social relationships, family, open data, necessity condition analysis

## Abstract

The Social Survey of Andalusia is an instrument for monitoring the Andalusian reality developed by the regional government of Andalusia, whose dataset is open access to the citizens. The 2022 edition included questions related to happiness, social relations as well as socio-demographic factors. Based on this dataset, the present study aimed to analyse the relationship between socio-demographic factors and people’s experiences of happiness. It also set out to explore which factors might be indispensable for experiencing moments of happiness as measured in the survey. A sample of 4,968 cases was gotten, conducting a descriptive analysis, a logit regression in complex samples, and a Necessary Conditions Analysis. The results found two very different social profiles in terms of the experience of happiness, determined by age, sex, educational level and economic status. However, neither factor proved to be a necessary condition for happiness. Both conclusions should be taken into consideration in any socio-community intervention.

## 1 Introduction

The Andalusian Social Survey (ESA) of 2022^1^ provides a comprehensive analysis of moments of happiness, social and family interaction, health concerns, and sociodemographic issues in Andalusia. This survey aligns with scientific evidence that highlights the relationship between affective responses, sociability, and health (1–3). In this sense, the ESA is an opportunity to assess the Andalusian population’s happiness and its relation to socialization.

Happiness, a complex concept, has been analyzed in various contexts including philosophy, psychology, physics, and art, leading to diverse interpretations (worldhappiness.report). It’s often compared or contrasted with concepts like virtuous life, subjective well-being, and life satisfaction (4, 5). Happiness is understood as an emotional expression resulting from personal appraisal linked to feelings (6, 7).

Research consulted indicate that happiness is influenced by cultural factors (8, 9) and positively correlates with extroversion (10). Studies show a reciprocal relationship between happiness and social behavior (11, 12), with people feeling happier in the company of acquaintances, friends, or family (13). Experimental studies have shown that improving socialization skills enhances happiness (14), suggesting that more social interaction increases self-reported happiness.

However, measuring happiness has methodological challenges (15). Questionnaire-based assessments should be interpreted carefully, considering participant bias (16). Moreover, well-being and health are correlated with happiness (17). Studies suggest a direct link between social relationships and physical and psychological health (3, 18–20), indicating benefits from emotional control and stress reduction due to socialization.

This study, justified by ESA survey data and scientific interest in happiness, aims to investigate the impact of social interaction on Andalusians’ self-reported happiness. The research also seeks to determine if specific factors are “necessary conditions” for happiness in the Andalusian population. This approach is influenced by the work of Martínez-Falcó et al. (21) and their bibliometric analysis on happiness management, underscoring its social relevance. The study’s objectives are to analyze the role of social interaction in Andalusians’ happiness and to explore the essential conditions for experiencing happiness.

## 2 Materials and methods

### 2.1 Design and sampling

This research began with a survey study based on the Andalusia Social Survey 2022 (ESA) conducted by the Institute of Statistics and Cartography of the regional government of Andalusia (IECA). This survey provide detailed information on how the population of Andalusia interacts with their environment and participates in various groups. Aspects such as new contacts, participation in associations, the impact of new technologies on social network creation, among other factors, are explored. ESA employs a sampling procedure consisting of a simple random stratified sample by province, taking into account the degree of urbanization. Data collection was carried out in the eight provinces that make up the Andalusian region. The degree of urbanization was specified by categorizing urban areas into three categories, depending on the number of residents: rural area, intermediate area, and urban area. The selection units were the residents in the 24 resulting groups (8 provinces by 3 urban categories). The sampling frame came from the Longitudinal Database of the Andalusian Population as of January 1, 2021.^2^

For the collection of cases, a proportional allocation was made according to the size of the provinces. Subsequently, a proportional allocation was established according to the population size within each province. As a result of the whole process, an effective sample of 4,968 participants was obtained, with a theoretical size of 8,750 people, whose characteristics are presented in Table 1. Data collection took place from April to July 2022. 83.65% of the cases were carried out through telephone procedures (CATI), while 16.4% were carried out via the Internet (CAWI) with a global response rate of 56.8% as reported by the Institute of Statistics and Cartography of Andalusia.^3^

**Table 1:** Sample characteristics.

Variable	Frequency
Sex	Male: 48.86%
	Female: 51.14%
Age	48.107 (S.E. = 0.437)
Nationality	Spanish: 90.30%
Household type	Couple with cohabiting children: 48.99%
	Couple without cohabiting children: 24.26%
	Single-person: 12.34%
	Single parent: 9.48%
Employment situation	Employed: 49.82%
	Unemployed: 10.11%
	Retired or similar: 22.48%
Educational level	Without formal studies: 7.12%
	Primary: 6.65%
	Secondary and VET: 62.17%
	University students: 21.56%
	Others: 2.41%
Main occupation	Services: 13.66%
	Technicians: 11.86%
	Elementals: 8.03%
Income	No income: 0.43%
	€450 or less: 2.83%
	From €451 to €900: 16.91%
	From €901 to €1,600: 30.54%
	From €1,601 to €2,500: 20.49%
	€2,501 or more: 19.56%

Consult full names of the categories in annexes. Percentages of the totals answered, the rest of the percentage corresponds to others, not applicable or not answered.

### 2.2 Instrument

The ESA utilizes a questionnaire whose methodological and construction characteristics can be consulted on IECA website.^4^ The ESA is structured into five blocks of questions: Block I on personal contacts; Block II on neighborhood relationships; Block III, questions about social relationships and networks of needs; Block IV with questions about citizen participation. These blocks are combined with sociodemographic data: age, sex, income level, educational level, and household type. The questionnaire combines multiple-choice, dichotomous and polytomous items. For this study, items linked to the constructs of happiness and social interactions were selected. Sociodemographic information was also taken into account (see Supplementary material).

Regarding happiness, the survey included the following question: “Could you tell me what makes you happy?” This question had 10 response options, where a maximum of 3 could be chosen. The initial results conducted by the Institute of Statistics and Cartography itself indicated that the most chosen options were: relationships with family, relationships with friends, and physical health (see Footnote 1). In the research presented here, only these variables were considered in relation to happiness.

### 2.3 Process and analysis

#### 2.3.1 Procedure and analysis

Initially, an analysis of the data from the ESA, available on the website of the Institute of Statistics, was carried out. The first step was to identify the sources or moments considered by the surveyed individuals to bring them the greatest happiness. In the study, these moments were treated as dependent variables.

The ESA was developed according to complex sampling procedures, as indicated by the Institute of Statistics and Cartography of Andalusia in the technical datasheet. The sampling design weights were calibrated using reweighting techniques, allowing for adjustments of the estimates to match the demographic information from the Longitudinal Database of the Andalusian Population. The weighting of each case was included in the “fep” variable of the dataset.

A descriptive analysis was applied to the collected data, estimating average values and corresponding response proportions for the population. Likewise, an exploratory analysis of the estimates of sources of happiness and social relationships was carried out according to the different population segments (by sex, age, educational level, profession, and income). Subsequently, a logit regression analysis for complex samples was performed on the previously defined dependent variables. In the face of a possible over-dispersion in the models, an approximation based on the quasi-binomial distribution was used (22). It is important to note that a stepwise forward and stepwise backward procedure was followed in model selection, and only the most notable final results are presented in the findings.

After the regression analysis, the Necessary Condition Analysis (NCA) by Dul’s (23) was developed. This identifies indispensable factors for a result, unlike linear regression where a single condition might suffice. This approach determines if the absence of a condition prevents a result, although its presence alone does not guarantee it, indicating other conditions may also be necessary.

The NCA analysis was structured in three stages:

- Hypothesis formulation based on essentiality: to determine whether the presence or absence of a variable is crucial for the manifestation of another variable.
- Estimation of the impact size using the CE_FDH method. The assessment was aligned with the guidelines of Dul (23), which categorize impacts as: no substantial (less than 0.1), moderate (0.1–0.3), substantial (0.3–0.5), or extremely substantial (greater than 0.5).
- Determination of the statistical relevance of the impacts through a bootstrap technique.

All analyses were conducted with R version 4.2.1 (24) including the Survey library version 4 (25). A statistical significance level of 0.01 or lower was always used for decision-making (N.C. = 99%).

## 3 Results

Starting with a descriptive analysis of the variables related to moments of happiness, the analysis revealed a dominant trend toward family relationships as the main source of happiness, with 91.6% of participants identifying it as such. Friendships and health (both physical and appearance) followed in importance with percentages of 46.6 and 41.4%, respectively. Romantic relationships (37.1%) and leisure activities (25.6%) were also considered substantial. Conversely, material possessions and work were less represented, with only 2.46 and 1%, respectively. Based on this data, the rest of the results focus on the three most frequently mentioned moments of happiness: family, social relationships, and health-related topics.

Regarding indicators of sociability level, 27.16% of participants reported contacting between 5 and 9 people in terms of daily interaction, either face-to-face or digitally. The categories of 10–19, less than four, 20–49, and 50 or more people accounted for 25.16, 16.08, 17.82, and 13.06%, respectively. In terms of face-to-face interactions, 34.73% of respondents saw all their daily contacts in person, followed by “most” (27.90%) and “about half” (21.62%). A 15.42% reported low daily face-to-face interaction.

An item of interest is the one that asks about the quality of family relationships. A 94.28% of participants described their relationships as good or very good. 4.82% rated them as fair, and only a small percentage (0.5%) considered them bad or very bad. It is relevant to note that 0.39% reported not having such relationships.

On the other hand, statistically significant differences were observed regarding sex in relation to happiness derived from sharing moments with family [Chi-squared (df = 1) = 23.51; *p* < 0.001]. Specifically, 47.7% of women reported happiness in these moments, compared to 43.7% of men.

When assessing differences by age, variations were found, although of moderate statistical relevance [Chi-squared (df = 3) = 21.40; *p* = 0.002]. The over-59 age segment reported the highest proportion of happiness with their family. They are followed, in descending order, by the age groups of 16–35 years (23.9%), 35–47 years (21.2%), and 47–59 years (20.3%). A lower proportion of happiness is noted in the middle-aged adult group.

No significant differences were observed in relation to family happiness when considering the educational level, employment, or income.

The second moment contributing most to happiness is the one shared with friends. Differences were found regarding sex in happiness associated with sharing moments with friends [Chi-squared (df = 1) = 13.247; *p* < 0.001]. 23.9% of men compared to 22.4% of women expressed happiness in these contexts. By age groups, those aged 16–35 years reported the highest degree of happiness (16.3%), followed by the group aged 35–47 years (10.5%), while older groups reported lower frequencies with highly significant differences Chi-squared (df = 3) =187.95 (*p* < 0.001).

Noticeable variations were found based on the level of education [Chi-squared (df = 9) = 173.02; *p* < 0.001], particularly distinctive between primary and secondary education levels. The details and their specifications are presented in Table 2.

**Table 2:** Percentages of happiness states among friends.

	YES, I am happy	NO, I’m not happy
**Educational level**		
0 = Lower than primary education	1.89%	5.22%
1 = Primary education	2.01%	4.64%
2 = First stage of secondary education and similar	11.97%	17.40%
3 = Second stage of secondary education and similar	11.96%	10.88%
4 = Non-higher post-secondary education	0.07%	0.02%
5 = Higher level vocational training, plastic arts and design and sports studies and equivalent; own university degrees that require a high school certificate, of an equal or greater duration of 2 years	5.45%	4.52%
6 = University degrees of more than 240 ECTS credits, university diplomates/junior college graduates, own university degrees as an expert or specialist, and similar	5.26%	4.64%
7 = University degrees of more than 240 ECTS credits, bachelor’s degrees, master’s degrees and specialties in Health Sciences through the residency system, and similar	7.05%	5.62%
8 = Doctoral studies	0.47%	0.40%
**Profession**		
0 = Military occupations	75.46%	24.54%
1 = Directors and managers	47.77%	52.23%
2 = Technicians and scientific professionals and intellectuals	58.23%	41.77%
3 = Technicians, support professionals	55.28%	44.72%
4 = Accounting, administrative and other office employees	52.18%	47.82%
5 = Catering, personal, protection and sales service workers	43.35%	56.65%
6 = Qualified workers in the agricultural, livestock, forestry and fishing sectors	43.72%	56.28%
7 = Skilled workers from the manufacturing industries and construction (except installation and machinery operators)	43.95%	56.05%
8 = Facility and machinery operators, and assemblers	45.02%	54.98%
9 = Elementary occupations	42.57%	57.43%
**Income**		
€0.00	45.43%	54.57%
<450€	38.82%	61.18%
€451–€900	35.87%	64.13%
€901–€1,600	44.93%	55.07%
€1,601–€2,500	52.39%	47.61%
€2,501–€3,000	53.08%	46.92%
3,000 < €	56.19%	43.81%

As for the occupation, there were statistically significant differences [Chi-square (df = 9) = 52.662; *p* < 0.001]. Table 2 presents these results, highlighting the divergent perception in the military career in comparison to other occupations, as well as notable trends among technicians, professionals, and office employees.

Regarding the income level, there were also statistically significant differences concerning friendships [Chi-square (df = 8) = 86.023; *p* < 0.001]. In Table 2, it is observed that respondents who consider friendship as a source of happiness the most are those with higher incomes (over €1,600 per month).

The third moment of happiness is linked to health and physical condition. Significant differences were observed according to sex in relation to happiness derived from health and physical well-being, Chi-square (df = 1) = 18.809 (*p* < 0.001). Women reported this factor as a reason for happiness to a greater extent (44.4%) compared to men (38.3%).

When evaluating the relationship with age, substantial differences were found [Chi-square (df = 3) = 65; *p* < 0.001]. The group aged 47–59 years showed the highest percentage considering health and physical condition as a source of happiness (47.5%), followed by those over 59 years (44.6%), the group aged 35–47 years (41.5%), and finally, those under 35 years (32.7%).

No statistically significant differences were recorded in relation to educational level, main occupation, or income level.

Another set of items corresponds to the number and quality of social relationships. The analysis revealed interesting patterns in the number of daily contacts reported by respondents. Women tend to report a medium range of daily contacts (1–9 contacts) more frequently than men. Conversely, a notable percentage of men (7.8%) reported more than 100 daily contacts, suggesting a higher level of social interaction in this group. The differences in the number of daily contacts between sexes were statistically significant [Chi-square (df = 6) = 63.022; *p* < 0.001].

Significant differences were also found with respect to age [Chi-square (df = 18) = 501.42; *p* < 0.001]. People aged 59 years or less tend to interact with a range of 10–19 individuals on a daily basis. On the contrary, those over 59 years of age reported that 64.32% maintained fewer than 10 daily contacts.

Similarly, there are statistically significant differences in the number of interactions according to the educational level. The analysis revealed notable differences in the number of daily interactions in relation to the level of education, Chi-square (df = 54) = 343.69 (*p* < 0.001). Specifically, people with a more basic educational level tend to report fewer daily contacts. Those with a medium educational level, such as secondary education, usually interact with a range of 10–19 people. However, individuals with advanced education levels tend to have more contacts, with frequencies that vary between 20 and 49 daily interactions. It is noteworthy to mention that those categorized under an educational level of “other” predominantly interact with a range of 5–9 people.

There are also statistically significant differences concerning the profession exercised [Chi-square (df = 54) = 168.86; *p* < 0.001]. Specifically, military personnel report frequent interactions, usually between 20 and 49 people, which aligns with the nature of their responsibilities. In contrast, intellectual professionals, support technicians, office workers, service staff, manufacturing workers, and operators report a more varied range, with daily interactions that can range from 5 to 49 individuals. On the other hand, agricultural workers and those in basic occupations generally connect with a more limited spectrum, between 5 and 19 people daily.

Regarding daily interactions according to the level of monthly income, significant differences were identified in the number of daily interactions based on monthly income levels [Chi-square (df = 36) = 265.56; *p* < 0.001]. Specifically, a lower income level generally correlates with fewer contacts. However, it is notable to mention that 25.12% of individuals who do not report income indicate having more than 100 daily interactions. On the other hand, middle incomes tend to correspond with a medium number of contacts.

In the next phase of the analysis, the dependent variables were the moments of happiness with family, the ones of happiness with friends, and those related to health. Different logit models were analyzed to estimate to what extent the independent variables of interest may be impacting the dependent variables.

Concerning the moments of happiness with family, two models were proposed for this dependent variable (see Table 3). The results indicate that the variables significantly associated with happiness during family times are the quality of family relationships (termed “relation”) and sex. In contrast, neither the number of contacts nor age proved to have a statistically significant impact on the probability of perceiving family gatherings as moments of happiness.

**Table 3:** Logit regression models on moments of happiness.

Model	Model expression	AIC
Family 1	happy1 = −2.852* – 0.058 (np) + 0.768 (relationship)* – 0.007 (age) – 0.45 (sex_woman)*	2703.846
Family 2	happy1 = −3.535* + 0.781 (relationship)* – 0.445 (sex_woman)*	2704.693
Friendship 1	happy2 = −0.436 – 0.052 (np) + 0.086 (relationship) + 0.020 (age)* + 0.180 (sex_woman) – 0.108 (nestu)* – 0.001 (cpro) + 0.00(ing)	6568.087
Friendship 2	Happy2 = −0.573* + 0.021 (age)* + 0.185 (sex_woman)' – 0.112(nestu)*	6567.367
Health 1	happy6 = 1.234* – 0.033 (np) – 0.083 (relationship) – 0.010 (age)* – 0.254 (sex_woman)*	6,679,940
Health 2	happy6 = 0.933* – 0.009 (age)* – 0.236 (sex_woman)*	6682.128

* Probability of *t* < 0.001; ' Probability of *t* < 0.01.

It can be concluded that Model 2 reflects more accurately the reality for the dependent variable feliz1 (family as a source of happiness) based on its AIC value and the principle of parsimony (see Table 3). Regarding the variable feliz2, which records whether being among friends is a source of happiness, the developed models present similar AIC values (see Table 3), although Model 2 is the most parsimonious. In this model, it stands out again that the number of contacts does not seem to affect the probability of considering meetings with friends as a moment of happiness. On the contrary, this probability is directly linked to age and inversely to the level of education. Additionally, being a woman also increases this probability.

Lastly, moments of happiness related to health and physical condition are negatively linked with age and being female. Thus, the probability of considering this factor as a source of happiness is less likely with increasing age, while it is higher in the case of being male compared to being female.

Finally, an analysis of conditions was performed based on the results of the previously conducted linear regression. A correlation was initially made between the dependent variables (states of happiness with family, with friends, and in relation to health) and the independent variables identified in the regression models. The correlations indicated that:

- The state of happiness with family and social relationships had a Pearson correlation of 0.203.
- The state of happiness with friends was correlated with age (Pearson Corr. of 0.223) and educational level (Pearson Corr. of −0.155).

The remaining correlations exhibited lower coefficients. From this, the following conditioning hypotheses were defined:

- Hypothesis of Model 1: The presence of a relationship is a necessary condition for “happy1” (happiness in family moments) to occur.
- Hypothesis of Model 2: The presence of age is a necessary condition for “happy2” (happiness in moments with friends) to occur.
- Hypothesis of Model 3: The absence of “nestu” (educational level) is a necessary condition for “happy2” not to appear.

The ce_fdh method was applied to calculate the effect of Possible Conditioning, yielding a value of 0 in all cases, indicating that no variable can be considered a necessary condition for the manifestation of states of happiness.

## 4 Discussion

Starting in an orderly manner with the experiences of moments of happiness, the results of the current study highlight the importance of family relationships for the Andalusian population, with older individuals experiencing a higher state of happiness with these relationships. This observation aligns with previous findings that emphasize social support in older adults as a prominent factor of well-being (26).

Regarding the age of the participants, the results clearly indicate that middle age emerges as the segment that perceives the least family happiness. This phenomenon may be due to the professional and domestic demands typical of this life stage (27), suggesting that these responsibilities may attenuate the enjoyment of family moments. However, this interpretation requires a more detailed analysis.

In the same vein, the results show that women express the most happiness in the family. Although this coincides with previous studies regarding overall well-being (28), it contradicts the conclusions of Caballero and Sánchez (29), whose results found no differences between sexes in a sample from the Autonomous Community of Madrid (regional government of Madrid in Spain).

In this regard, it must be considered that cross-cultural studies have revealed differences between countries and cultures (30–33), which raises the question of whether the Andalusian population harbors distinctive cultural peculiarities, or whether underlying socioeconomic causes can be identified to explain these results.

The second set of results are related to the state of happiness that arises from the company of friends. The study reveals that young people are the ones who value these bonds the most, which is consistent with the evolutionary nature of relationships during this stage (34). As Van Zalk et al. (35) explain, during the young adult stage, friendships and peer social relationships can provide valuable emotional support, as well as the opportunity to share interests and common activities, acting as self-reinforcement of one’s own social behavior. In fact, peer interactions during these years seem to be linked to the experience of life satisfaction, mental health, and mental well-being during young adulthood, as research in the area has shown (36).

Regarding profession, the military collective stands out in the professional field, emphasizing friendship as essential for their well-being, corroborating previous research (37).

As for the number of social contacts, the results clearly show that older individuals have fewer daily contacts. This may be due to multiple factors, among others like the decrease in mobility of this segment of the population (an explanation that would only be valid for those individuals with some kind of motor deficit), as well as the reduction of the usual social circle (38, 39).

Within these findings, a complex relationship between educational and economic level with happiness is evident, such that it has been found that individuals with low resources and education exhibit fewer experiences of happiness among friends. In this context, it is noteworthy that previous studies have found that people with lower educational level and economic resources have a smaller number of social contacts (40). These two facts lead to the question of whether belonging to the segment of the population with lower economic capacity and academic training implies having to face certain challenges in accessing social and work spaces where social interactions are richer. And in any case, what impact this has on the well-being of the individual. This observation not only highlights a social reality, but it is also an invitation to question and explore in more depth the dynamics between education, economy, and social relationships.

Regarding the dimension of health and physical condition, it is women, or people with higher socioeconomic status, who express the most happiness. This result contradicts previous studies where no differences were found between men and women in relation to happiness (41, 42) supporting those that do find differences between sexes (see (43)). However, these findings demand more detailed scrutiny, considering the definitional and methodological discrepancies between studies.

In summary, the study coincides with a large part of the consulted literature, where the complexity of happiness as an individual experience is highlighted (see the review by (44)). In this regard, any social intervention must take into account the complexity of the construct itself.

However, all the above is called into question by the Necessary Conditions Analysis. Despite the regression models and initial correlations that suggested the relevance of certain variables as necessary conditions for the emergence of states of happiness, the results of the analysis clearly contradict these expectations. Specifically, the effect size for all the proposed variables was null, which indicates that none of the variables examined can be considered a necessary condition for happiness in the analyzed contexts.

This research prompts a reevaluation of the conventional understanding that social relationships are a primary source of happiness. It suggests a more nuanced exploration of other potential variables and interactions affecting happiness. Echoing Trotsuk and Grebneva (45), happiness is recognized as an interdisciplinary subject, characterized by its conceptual and methodological diversity.

The study uncovers a multifaceted relationship between various factors and the perception of happiness among the Andalusian population. Specifically, it finds that older individuals, particularly within family contexts, report higher happiness levels. This demographic tends to view social support as a crucial well-being component as they age. For women, family relationships emerge as substantial contributors to emotional well-being. In contrast, younger people often derive happiness from friendships, presenting an interesting variation in sources of contentment. Furthermore, the study notes that health and socioeconomic status play pivotal roles in influencing happiness, with women and those of higher socioeconomic status reporting greater levels of happiness, particularly in these areas.

A fundamental conclusion of the study is that there are two major sociodemographic profiles associated with the presence and absence of happy experiences:

- Older individuals with a higher socioeconomic level who get along well with family and maintain daily contact with a moderate to high number of people in their environment tend to have higher happiness indexes. They often have professions in fields that allow them to have good social relationships, both in quality and quantity, such as the army, technology, business, education, or health.
- Less happy individuals tend to be those aged between 35 and 59 with a low socioeconomic level, a low number of daily contacts, deficient family relationships, and whose profession also does not allow them to improve their emotional experience.

Another relevant conclusion is that, despite these results and the concurrence with previous studies, there are not sufficient analytical arguments to consider that none of the factors linked to happiness is an indispensable condition for experiencing it.

In conclusion, the ambiguity surrounding happiness reveals that we are still far from understanding it fully, underscoring from here the need to delve deeper into its multifaceted nature.

## Data availability statement

Publicly available datasets were analyzed in this study. This data can be found at: https://www.juntadeandalucia.es/servicios/estadistica-cartografia/actividad/detalle/175352.html.

## Ethics statement

Ethical review and approval was not required for the study on human participants in accordance with the local legislation and institutional requirements. Written informed consent from the patients/ participants or patients/participants’ legal guardian/next of kin was not required to participate in this study in accordance with the national legislation and the institutional requirements.

## Author contributions

AM-T: Conceptualization, Data curation, Formal analysis, Investigation, Methodology, Project administration, Resources, Software, Supervision, Validation, Visualization, Writing – review & editing. CS: Conceptualization, Writing – review & editing. JM-T: Conceptualization, Writing – original draft, Writing – review & editing.

## Footnotes

^1^ https://www.juntadeandalucia.es/institutodeestadisticaycartografia/encsocial/2022-relaciones-sociales/notaprensa.htm

^2^ https://www.juntadeandalucia.es/servicios/estadistica-cartografia/actividad/detalle/175352.html

^3^ http://www.juntadeandalucia.es/institutodeestadisticaycartografia/digitalizacion-sociedad-digital/index.htm

^4^ https://www.juntadeandalucia.es/institutodeestadisticaycartografia/dega/sites/default/files/docs/121-encuesta-social-2022-relaciones-sociales-metodologia.pdf
