# Supplementary material for: Demographic determinants of happiness in Andalusia: insights from the 2022 social survey data
Source: Front Public Health. 2024 May 2;12:1338494. doi: 10.3389/fpubh.2024.1338494 (PMC11096586; doi:10.3389/fpubh.2024.1338494)
Supplement: Supplementary file 1 [file Table_1.docx]

# Supplementary materials

**Table .Variables used from ESA**

| Items |  |
| --- | --- |
| Below are the analyzed items. In parentheses is the variable code, followed by the item number or category code. The original ESA questionnaire can be found at https://www.juntadeandalucia.es/institutodeestadisticaycartografia/encsocial/2022-relaciones-sociales/metodologia/cuestionario.pdf |  |
|  |  |
| Block of questions about "personal contacts" |  |
| 1. Could you tell me what makes you happy? (up to three responses) |  |
|  | (happy1) - Relationships with family: YES NO |
|  | (happy2) - Relationships with friends: YES NO |
|  | (happy6) - Your physical health and/or appearance: YES NO |
|  |  |
| **(np) 2.** Please tell me, on a typical weekday, approximately how many people do you have contact with, regardless of whether you know them or not. We mean people you talk to or exchange messages with, whether in person, by phone, online, or through any other means. |  |
|  | 1. None |
|  | 2. 1-4 people |
|  | 3. 5-9 people |
|  | 4. 10-19 people |
|  | 5. 20-49 people |
|  | 6. 50-99 people |
|  | 7. 100 or more people |
|  | 98. Don't know |
|  | 99. No answer |
| Observation: If np=1 => 4 (If np<>1 => 3) |  |
|  |  |
| **(relationship) 6**. In general, do you consider the relationships you have with your family to be...? (complete family network) |  |
|  | 1. Very good |
|  | 2. Good |
|  | 3. Normal |
|  | 4. Bad |
|  | 5. Very bad |
|  | 6. There is no relationship |
|  |  |
| Block of questions about "sociodemographic characteristics" |  |
| **(nestu) 28**. What is the highest level of education you have completed? |  |
| Highest level of completed education: ____________ |  |
| NOTE. Tabulation: |  |
|  | 0=Less than primary/ |
|  | 1=Primary education/ |
|  | 2=First stage of secondary education and similar/ |
|  | 3=Second stage of secondary education and similar/ |
|  | 4=Post-secondary non-higher education/ |
|  | 5=Higher vocational education, plastic arts and design, and sports training equivalent to university degrees requiring a high school diploma, lasting 2 years or more/ |
|  | 6=University degrees of up to 240 ECTS credits, university diplomas, university titles of expert or specialist, and similar/ |
|  | 7=University degrees of more than 240 ECTS credits, bachelor's degrees, master's degrees, and specialties in Health Sciences through residency, and similar/ |
|  | 8=Doctoral studies/ |
|  | 9=Cannot be coded (others) |
|  |  |
| **(con) 30.** In your main job or in your last job, what is/was the occupation, profession, or trade you are/were engaged in? (Base=1,2,3,4,5,6,7,8 in Q.29) |  |
| Occupation: ____________ |  |
| NOTE. Tabulation: |  |
|  | 0=Military occupations/ |
|  | 1=Directors and managers/ |
|  | 2=Technicians and scientific and intellectual professionals/ |
|  | 3=Technical and support professionals/ |
|  | 4=Accounting, administrative, and other office employees/ |
|  | 5=Workers in restaurant services, personal services, protection, and sales/ |
|  | 6=Skilled agricultural, livestock, forestry, and fishing workers/ |
|  | 7=Skilled workers in manufacturing industries and construction (except machinery operators and assemblers)/ |
|  | 8=Machinery operators and assemblers/ |
|  | 9=Elementary occupations/ |
|  | -1=Not stated/ |
|  | -9=Not applicable |
|  |  |
| **(ing) 31.** Tell me in which interval, of those I will read to you next, are the monthly net incomes of all members of your household. |  |
|  | 1. No income |
|  | 2. Less than 450 euros |
|  | 3. 451 to 900 euros |
|  | 4. 901 to 1,600 euros |
|  | 5. 1,601 to 2,500 euros |
|  | 6. 2,501 to 3,000 euros |
|  | 7. More than 3,000 euros |
|  | 98. Don't know |
|  | 99. No answer |
